# The Fast and the Furious: Low-Risk Chest Pain and the Rapid Rule-Out Protocol

**DOI:** 10.5811/westjem.2016.12.32676

**Published:** 2017-02-27

**Authors:** Maite Anna Huis in ’t Veld, Louise Cullen, Simon A. Mahler, Barbra E. Backus, Zachary D.W. Dezman, Amal Mattu

**Affiliations:** * University of Maryland Medical Center, Department of Emergency Medicine, Baltimore, Maryland; † Royal Brisbane and Women’s Hospital, Department of Emergency Medicine, Brisbane, Queensland, Australia; ‡ Wake Forest School of Medicine, Department of Emergency Medicine, Winston-Salem, North Carolina; § Medisch Centrum Haaglanden, Department of Emergency Medicine, The Hague, Netherlands; ¶ University of Maryland School of Medicine, Department of Emergency Medicine, Baltimore, Maryland

## Abstract

Accelerated diagnostic pathways (ADP) have been designed to identify low-risk chest pain patients in the emergency department. This review article discusses the Asia-Pacific Evaluation of Chest Pain Trial (ASPECT) score, the Accelerated Diagnostic Protocol for Chest Pain Trial (ADAPT) score, the Emergency Department Assessment of Chest Pain Score (EDACS), the HEARTScore and the HEART pathway. These ADPs have been validated in various studies and aid the emergency provider with identifying the low-risk chest pain patient who is appropriate for discharge home, while at the same time highlighting those patients who would benefit from further in-patient work up. These approaches should be paired with patient input and shared decision-making strategies.

## INTRODUCTION

Chest pain is one of the most frequent complaints of patients presenting to the emergency department (ED). Approximately 10–20% of patients who present to the ED with chest pain are suffering from an acute coronary syndrome (ACS), requiring early intervention and treatment.^1^ In the remaining 80% of patients chest pain symptoms are explained by other, often not life-threatening, conditions. Distinguishing patients suffering from ACS from those who are not based on their chest pain history and physical examination is difficult as no history or examination variables have sufficient predictive value to rule in or rule out ACS, if considered in isolation. Admission for further workup of chest pain patients for the evaluation of ACS is costly, time consuming and places patients at risk of adverse events during their evaluation. Early discharge is also not without risk, as up to 2–5% of patients with ACS are inappropriately discharged from the ED every year.^2^ Missed ACS remains a top malpractice claim in the United States. These current care patterns of over- and under-testing demonstrate that the low-risk chest pain evaluation is a diagnostic dilemma for emergency clinicians.

The American College of Cardiology and American Heart Association (ACC/AHA) have developed guidelines in an attempt to standardize the approach to patients with chest pain. The 2010 and 2014 guidelines recommend the use of the Global Registry of Acute Coronary Events (GRACE) score or thrombolysis in myocardial infarction (TIMI) risk score as part of the initial evaluation for possible ACS. However, neither TIMI nor GRACE was designed for ED chest pain risk stratification. The TIMI score was designed to be applied to patients with unstable angina or non ST-elevation myocardial infection (NSTEMI) to determine their risk for 14-day mortality, new or recurrent acute myocardial infarct (AMI) or severe recurrent ischemia requiring urgent revascularization.^3^ The GRACE score was developed to risk stratify patients with confirmed ACS to estimate their in-hospital, six-month and three-year mortality.^4^ While these scores were subsequently validated in an ED setting, they lack the sensitivity needed to identify a low-risk population capable of safe early discharge from the ED.^5^

Accelerated diagnostic pathways (ADP) were developed to provide guidance to emergency clinicians to determine the level of risk for patients with possible ACS and support appropriate testing for each patient with chest pain. The goal of ADPs is to identify the very low-risk patients for early discharge, while at the same time identifying those patients more likely to benefit from stress testing and angiography. ADPs are starting to appear in ACC/AHA guideline updates. A more detailed description of the most commonly used ADPs is found below.

## ACCLERATED DIAGNOSTIC PATHWAYS

### ASPECT

The ASia-Pacific Evaluation of Chest Pain Trial (ASPECT) aimed to prospectively validate the safety of a predefined two-hour ADP to identify ACS patients. The study was conducted in 14 EDs in nine countries in the Asia-Pacific region.^6^

Those with a TIMI risk score of 0, no new ischemic changes on the electrocardiogram (ECG) and a negative 0- and 2-hour point-of-care biomarker results were deemed low risk and eligible for early discharge. Primary endpoint was major adverse cardiac events (MACE) within 30 days. MACE was defined as death (not clearly non-cardiac), cardiac arrest, an emergency revascularization procedure, cardiogenic shock, ventricular arrhythmia needing intervention, high-degree atrioventricular block needing intervention and acute myocardial infarction. A total of 3,582 patients were enrolled, of which 352 (10%) were considered low risk. Within the low-risk group there were three (0.85%) MACE. The ASPECT ADP had a 99.3% (95% confidence interval [CI] [97.9 – 99.8]) sensitivity with a negative predictive value of 99.1% (95% CI 98–100) for MACE.

### ADAPT

The Accelerated Diagnostic Protocol for Chest Pain (ADAPT) trial was a prospective observational study from the same investigators as the ASPECT trial.^7^ In this trial, 1,975 patients were enrolled in two of the ASPECT centers to identify low-risk patients suitable for discharge after application of an ADP incorporating sensitive/contemporary troponin assay results, with TIMI score and ECGs (Tables 1 and 2). The investigators found that 392 patients (20%) were classified as low risk. One of 392 patients (0.25%) had a MACE. The ADAPT score was found to have a sensitivity of 99.7%, specificity of 23.4%, negative predictive value of 99.7% and positive predictive value of 19% for MACE.

In the Advantageous Predictors of Acute Coronary Syndrome Evaluation (APACE) validation study, patients were classified as low risk if they had a TIMI score of 0 or 1 (0 only in the ADAPT trial), a non-ischemic ECG and normal 0- and 2-hour high sensitivity troponin measures (Table 1).^8^ Of the 909 patients enrolled, 40% were identified as low risk. This validation study found a sensitivity of 99.4% (95% CI [96.5 – 100]), NPV 99.7% (95% CI [98.4 – 100]).

### EDACS

The Emergency Department Assessment of Chest Pain Score (EDACS) is the first emergency medicine-based risk score derived from clinical data and was developed as a chest pain score to identify patients safe for early discharge. The derivation cohort consisted of patients enrolled in the ADAPT study.

The EDACS score was incorporated into an ADP, where low risk was identified as an EDACS score < 16, no new ischemia on ECG and a negative 0- and 2-hour troponin. In this observational cohort patients who met these criteria were identified who would have been safe for discharge home without further workup. The derivation cohort (1,974 patients) and the validation cohort (608 patients) identified 40–50% of patients as low risk. The sensitivity was 99% (95% CI [96.9–99.7]) for MACE. EDACS has been validated in a randomized trial comparing EDACS to ADAPT.^9^ In this study more patients were identified as low risk by EDACS compared to ADAPT, and no patients identified as low risk had a 30-day MACE event. However, in the first U.S. validation study EDACS had lower sensitivity for MACE.^10^

### HEARTScore

The HEARTScore was developed to score predictors of primary end points based on clinical experience and previous medical literature.^11^ Predictors included history (H), electrocardiography (ECG) (E), Age (A), Risk factors (R) and Troponin (T), forming the HEART score. Each of the five factors is scored with 0, 1, or 2 points (Table 4). Patients were followed for six weeks for a primary end point of major adverse cardiac event (MACE), including AMI, primary coronary intervention (PCI), coronary artery bypass graft (CABG) or death.

In the first retrospective validation study 122 patients presented to the ED with chest pain. Results are presented in Table 5. One (2.5%) of the 39 patients with a low HeartScore (0–3) had a MACE, requiring CABG. This was compared to 12 of 59 (20.3%) patients with a HeartScore of 4–6, and 16 of 22 (72.7%) of patients with a HeartScore of 7–10 points that reached an endpoint. Two deaths occurred in the study; both patients had a HeartScore of eight. After this small retrospective study, a multicenter retrospective study was performed.^2^ In this study 34% of patients were identified as low risk, with a risk of MACE of 0.99%. The results of this study are presented in Table 5. Both studies, however, were limited by their observational, retrospective design. Further validation was needed, and the same authors provided a prospective multicenter study.^5^ In this study the HeartScore was compared to the TIMI and GRACE scores. A total of 2,440 patients who presented to the ED with chest pain were enrolled in 10 Dutch hospitals. Outcomes measures were the same as the retrospective studies. The results of the HeartScore original study and validation studies are presented in Table 5.

Sixteen patients died (0.7%), 13 of whom died of a cardiac cause. One of these patients was in the low-risk HeartScore group, five were in the intermediate-risk group and seven in the high-risk HeartScore group. The C-statistics of the HeartScore when compared to TIMI and GRACE were as follows: HEART 0.83, TIMI 0.75, GRACE 0.70 (p<0.0001). This study provided additional support for use of the HeartScore as an ADP for low risk chest pain patients.

### HEART Pathway

While the HeartScore is predictive of MACE, many clinicians consider the 1.7% risk of MACE in a patient identified as low risk by the HeartScore to be too high. Furthermore, with the HeartScore it is possible to have a patient with a low-risk HeartScore, despite a positive troponin. The Heart pathway was designed to lower the missed MACE rate of the HeartScore below 1%, by separating the troponin results from the remaining “Hear” score and using two troponin measures (at 0 and 3 hours) instead of one. To be considered low-risk using the HeartScore pathway you must have a Hear(t) score of 0–3 and have both serial troponin measures less than the 99^th^ percentile upper-reference limit.

The first study to validate the HeartScore in the U.S. enrolled 1,070 chest pain patients in an observation unit and revealed that five patients with an NSTEMI had low-risk HeartScore.^13^ However, all of these patients had positive serial troponins. Use of the Heart pathway, with its serial troponins, was 100% sensitive for ACS and could have decreased observation stays by 80%. A secondary analysis performed on 1,005 participants in the Myeloperoxide in the Diagnosis of Acute Coronary Syndromes Study (MIDAS) found the Heart pathway to identify 20% of patients for early discharge with a 99% (95% CI [97%–100%]) sensitivity for ACS.^14^ The Heart Pathway Randomized Controlled Trial evaluated 282 patients and randomized them to the Heart pathway or usual care. Use of the Heart pathway increased early discharge by 21% (p=0.0002), median length of stay was decreased by 12 hours (p=0.013), and objective cardiac testing at 30 days was decreased by 12% (p=0.048), without any MACE events among patients identified as low risk.

## SHARED DECISION-MAKING

In recent years there has been growing attention to shared decision-making. Shared decision-making involves educating patients on their health risks, as well as the risks of testing, and discussing their treatment options. This is often done using a pictogram developed at the Mayo Clinic called the Chest Pain Choice.^15^ In the Chest Pain Choice Trial, a single-center randomized controlled trial, patients enrolled in the shared decision-making arm reported greater knowledge, less decisional conflict and feeling more engaged in the decision-making process when compared to those receiving usual care. Patients also decided less frequently to be admitted for further testing, with a 19% absolute difference (95% CI [6%–31%]).

## SUMMARY

The low-risk patient with chest pain can be a high-risk scenario for the emergency physician. Accelerated decision protocols have been designed to aid the emergency physician in decision-making with regards to assessment of these patients. The use of these ADPs can reduce cost, length of stay and risk of unnecessary testing in chest pain patients. It is important for all emergency physicians to be familiar with different ADPs, and to know their benefits and limitations. All of the above-described ADPs are validated choices for risk assessment of low-risk chest pain patients in the ED. UsTe of any of these ADPs should be considered within standard of care. The choice to select a specific ADP for use in the ED can be done on an institutional level or can be the choice of the individual practitioner. Within the authors’ (MH, AM, ZD) institution, the Heart pathway was implemented alongside a shared decision model for its high sensitivity, negative predictive value and ease of use. Shared decision-making tools may assist patients with acute chest pain and their providers to navigate difficult disposition decisions.

## Figures and Tables

**Table 1:** Low-risk patients as classified in the ASPECT, ADAPT and APACE trial.^6^,^7^,^8^

ASPECT	ADAPT	Modified ADAPT
Contemporary troponin, myoglobin and CK-MB negative at 0 and 2 hours	Contemporary troponin negative at 0 & 2 hours	High sensitive troponin negative at 0 & 2 hours
ECG without new ischemic changes	ECG without new ischemic changes	ECG without new ischemic changes
TIMI score 0	TIMI score 0	TIMI score 0 or 1

*ASPECT*, Asia-Pacific Evaluation of Chest Pain Trial; *ADAPT,* Accelerated Diagnostic Protocol for Chest Pain Trial; *APACE,* Advantageous Predictors of Acute Coronary Syndrome Evaluation; *CK-MB,* creatine kinase-MB; *ECG,* electrocardiogram; *TIMI,* thrombolysis in myocardial infarction.

**Table 2:** TIMI score.^3^

Age ≥ 65	+ 1
≥ 3 CAD (coronary artery disease) risk factors	+ 1
Known CAD (stenosis ≥ 50%)	+ 1
Aspirin use in past 7 days	+ 1
Severe angina (≥ 2 episodes in 24 hours)	+ 1
ECG ST changes ≥ 0.5mm	+ 1
Positive cardiac marker	+ 1

*TIMI,* thrombolysis in myocardial infarction; *ECG,* electrocardiogram

**Table 3:** Emergency Department Assessment of Chest Pain Score (EDACS).

Clinical characteristics	Score
Age	
18 – 45	+ 2
46 – 50	+ 4
51 – 55	+ 6
56 – 60	+ 8
61 – 65	+ 10
66 – 70	+ 12
71 – 75	+ 14
76 – 80	+ 16
81 – 55	+ 18
86 +	+ 20
Male sex	+ 6
Aged 18 – 50 years and either: known coronary artery disease or ≥3 risk factors	+ 4
Symptoms and signs	
Diaphoresis	+ 3
Radiates to arm or shoulders	+ 5
Pain occurred/worsened by inspiration	− 4
Pain is reproduced by palpation	− 6

**Table 4:** The HEARTScore for chest pain patients in the emergency department.^11^

HEARTScore		
History	Highly suspicious	2 points
	Moderately suspicious	1 point
	Slightly or non suspicious	0 points
ECG	Significant ST-depression	2 points
	Nonspecific repolarization	1 point
	Normal	0 points
Age	>= 65 years	2 points
	> 45 – <65 years	1 point
	<= 45 years	0 points
Risk factors*	>= 3 risk factors or history of CAD	2 points
	1 or 2 risk factors	1 point
	No risk factors	0 points
Troponin	>= 3x normal limit	2 points
	>1 – <3 normal limit	1 point
	<= normal limit	0 points

* Risk factors: diabetes mellitus, current or recent (<one month) smoker, diagnosed hypertension, diagnosed hypercholesterolemia, family history of coronary artery disease and obesity.

**Table 5:** HeartScore, risk of MACE within six weeks from ED presentation.

	Risk of MACE at 6 weeks in original study^11^	Risk of MACE at 6 weeks in validation study^5^
Low HeartScore (0 – 3)	2.5 % (1/39)	1.7% (15/870)
Intermediate HeartScore (4 – 6)	20.3 % (12/59)	16.6 % (183/1101)
High HeartScore (7 – 10)	72.7 % (16/22)	50.1 % (209/417)

*HeartScore* (history, ECG, age, risk factors, troponin); *MACE*, major adverse cardiac event; *ED,* emergency department
